# Book Review: Statistical Inference as Severe Testing

**DOI:** 10.3389/fpsyg.2019.00735

**Published:** 2019-04-05

**Authors:** Jose D. Perezgonzalez, Marcos Pascual-Soler, Juan Pascual-Llobell, Dolores Frias-Navarro

**Affiliations:** ^1^School of Aviation, Massey Business School, Massey University, Palmerston North, New Zealand; ^2^ESIC Business & Marketing School, Valencia, Spain; ^3^Department of Methodology of the Behavioral Sciences, Universitat de València, Valencia, Spain

**Keywords:** epistemology, logic, statistics, philosophy, methodology

Deborah Mayo uses the heuristic of a visit to the museum of inferential statistics to contextualize her philosophy of inference via severe testing; that is, of inferences based on claims having passed stringent statistical probes with highly reliable methods (Mayo, 2018, p. 10) so as to guard against the fallacies of acceptance and rejection. The visit to the museum covers six excursions (sections), 16 tours (chapters), 26 souvenirs (summaries), and 40 exhibits (detailed examples). It also juggles recurrent topics to keep in mind during the visit: deductive falsification, induction, probabilism, frequentist performance, and severity testing. The visitor needs beware that, while the entry ticket announces in big red bold font that this visit is for statistical inference, the entire experience is more accurately described by the small print, of relishing in historical and contemporary statistics wars.

The book sets its main goal as that of tackling foundational problems of statistical practice (p. xiv), and Mayo places it as a cornerstone of her philosophical-practical triangle for understanding statistical inference, together with her works *Error and the Growth of Experimental Knowledge* (Mayo, 1996), and *Error and Inference* (Mayo, 2010)^1^. The book delivers on that goal and seems particularly successful in tackling the task of demystifying pervasive frequentist myths, such as the fallacy of interpreting differently the “reliability” of significant results when sample sizes (p. 240) or effect sizes (p. 325) get larger, or the role of power in statistical inference (p. 324). Most importantly, the book sets itself apart by an overarching emphasis on the need to audit methods before fully engaging in inference (e.g., p. 94, 193), so much so that Mayo even dedicates two tours (chapters 4.III and 4.IV) to the auditing of sampling and model checking, respectively.

The book is, thus, insightful for readers who first come across Mayo's philosophy of statistics, although they will miss a compelling tutorial for severity testing, as the text often engages the statistics wars—even book reviewers get caught by the war theme more than by the inferential one; (e.g., Heard, 2018; Morey, 2018; Robert, 2019). Unfortunately, as far as the statistics wars are concerned, it seems that Mayo's book is both “talking to a [Bayesian] brick wall” and “preaching to the converted [frequentist].” The former because the arguments regarding probabilism and enumerative induction have equally been used by Bayesians as foundations for their own inferential approach, so they will hardly be convinced otherwise^2^. The latter because a frequentist already using Neyman-Pearson's or Fisher's tests will hardly need convincing of continuing the frequentist approach either, yet they may miss Mayo's demarcation between significance and severity, and risk falling back onto “it's all just significance testing.”

So, once we brush over the statistics wars, what does *Statistical Inference as Severe Testing* offer to the severe tester? It offers a sound and complete method for learning via falsification. Indeed, severity testing rests on Popper's falsificationist philosophy (p. 75), and enhances it by focusing on the *ad hoc* learning that we may gain from controlling and dispelling errors from our methods of enquiry (a.k.a., methodological falsificationism; p. 83). Inferential errors may be found at any, even multiple levels, in the link between data collection and theory (p. 87). Indeed, both formal and informal auditing are needed to make sure errors are envisaged, identified, and controlled before we are ready to substantiate an inference. Failing an audit, we have bad evidence, no test (p. 5). For good inferences we require both a genuine desire to find things out and, at minimum, weak severity: a sound attempt at ruling out ways a claim may be false (p. 5). For optimal inferences, however, we require strong severity: a stringent procedure highly capable of finding flaws in a claim if such flaws existed (p. 14).

Yet passing a stringent test is not enough for moving from weak to strong severity. It is the entire procedure which needs to be stringent: audited (p. 269), multi-tested (p. 99, 301), piecemeal testing to resolve instantiations of Duhem's problem (p. 83), formal and informal scrutiny (p. 109, 279), etc. The aim is to achieve lift-off (p. 15) via an argument from coincidence (p. 14) rather than because of frequentist performance in the long run. Indeed, it is not so much that three reliable scales are able to measure a weight discrepancy with a smaller margin of error over time but—and here is Mayo's insight—that they inform us with great probability if any of the scales is unsound now (p. 15). Upon finding no error despite the strong probes, we may infer that an error is absent; meanwhile the convergence of weight discrepancies allows for an argument from coincidence and lift-off: “an inference more reliable and precise than its premises individually” (p. 15). Thus, Mayo's severity is about methodological severity—about how well-tested a claim is (e.g., p. 10)—not about inferential severity *per se*—although data is evidence for a claim just to the extent that the claim has passed a severe test with such data (p. 108)—nor about the probability of a claim—whether the data renders the claim improbable (p. 10).

In brief, the book is insightful for those new to Mayo's philosophy of statistics and provides a thorough view of the statistics wars. For the concerned methodologist, it also demarcates severity tests from significance tests and power analyses, and provides an excellent—albeit at time confusing—framework of methodological falsificationism for better science.

## Author Contributions

JP initiated and drafted the review. MP-S, JP-L, DF-N, and JP revised and edited the manuscript. All authors approved the final version of the manuscript for submission.

### Conflict of Interest Statement

The authors declare that the research was conducted in the absence of any commercial or financial relationships that could be construed as a potential conflict of interest.

## Footnotes

^1^It also inherits Mayo's tendency to re-use her previous writing as long as it remains relevant, which makes the book less enlightening for readers knowledgeable of her previous work.

^2^Indeed, 22 years earlier Mayo had already written, “I do not hope to bring hard-core Bayesians around to my view, but I do hope to convince the large pool of tempered, disgruntled, and fallen Bayesians that a viable non-Bayesian alternative exists” (Mayo, 1996, p. xiii).
